# Impact of integrating out-of-hours services into Emergency Medical Services Copenhagen: a descriptive study of transformational years

**DOI:** 10.1186/s12245-022-00442-4

**Published:** 2022-08-25

**Authors:** Nienke D. Zinger, Stig Nikolaj Blomberg, Freddy Lippert, Thomas Krafft, Helle Collatz Christensen

**Affiliations:** 1 Emergency Medical Services Copenhagen, University of Copenhagen, Copenhagen, Denmark; 2 Department of Health, Ethics & Society, CAPHRI School of Public Health and Primary Care, Faculty of Health, Medicine and Life Sciences, Maastricht University, Maastricht, The Netherlands; 3 Danish Clinical Quality Program (RKKP), National Clinical Registries, Copenhagen, Denmark

**Keywords:** Integrated care, Emergency medical services, Out-of-hours medical services, Telephone helpline

## Abstract

**Background:**

Many emergency medical services and out-of-hours systems are facing an increasing demand for primary, ambulance, and secondary care services caused by population aging and a higher prevalence of long-term and complex conditions. In order to ensure safety and efficiency for future demands, many systems are changing their dispersed healthcare services towards a more integrated care system. Therefore, an evaluation of the production and performance over time of such a unified system is desirable.

**Methods:**

This retrospective quantitative study was performed with dispatch and financial accounting data of Copenhagen Emergency Medical Services for the period 2010–2019. Copenhagen Emergency Medical Services operates both an emergency number and a medical helpline for out-of-hours services. The number of calls to the emergency number, the centralized out-of-hours medical helpline, the number of dispatches, and the annual expenditure of the system are described for both the periods before and after the major reforms. Production of the emergency number and the centralized medical helpline were analyzed separately.

**Results:**

The average number of dispatches increased from 328 per 10,000 inhabitants in 2010 to 361 per 10,000 inhabitants in 2019. The newly initiated medical helpline received 533 calls per 10,000 inhabitants in its first year and 5 years later 548 calls per 10,000 inhabitants. A cost increase of 10% was observed in the first year after the reforms, but it decreased again to 8% in the following year.

**Conclusions:**

There is a population demand for a centralized telephone access point for (semi-)emergency medical services. A more integrated EMS system is promising for a sustainable healthcare provision for a growing population with complex healthcare demands and multi-morbidities.

## Key messages

*What is already known at this topic*

- Examples exist of nurse-staffed out-of-hours services, either as part of the primary care or as part of the hospital services, among others in countries such as the Netherlands and Ireland.

*What this study adds*

- This study describes the highly integrated out-of-hours service that is embedded in the emergency medical services as a single entry point to healthcare in Copenhagen, Denmark, which is unique in Europe in its kind.
- Out-of-hours services are an important gateway to the healthcare provision for citizens after normal opening hours of general practitioners and help to regulate the flow of patients to emergency departments. This study presents a description of the organization of the integrated services and the financial and patient-directed consequences.

*How this study might affect research, practice, or policy*

- Whenever a community is faced with a need to organize or reorganize the patients’ access to out-of-hours services and patients’ access to healthcare in general, there are only a few available descriptions of integrated systems existent. There is a large variety of services, ranging from those that rely on untrained dispatchers who handle the calls to systems where the out-of-hour calls are handled by highly trained medical doctors. It is important for each community to choose a system that strikes a balance between the quality delivered to the citizen on one hand and the availability of the necessary resources on the other hand.

## Background

As many healthcare systems at that time, the emergency medical services (EMS) and out-of-hours (OOH) primary care provision in the Copenhagen region, Denmark, consisted before 2014 of many separate care units that operated relatively independent from each other. Consequently, the citizens with healthcare needs could enter the system via many different access points. For example, entrances were established via the out-of-hours numbers of the citizens’ general practitioners (GPs), but also all emergency departments of the region’s hospitals and mental health centers had all different telephone numbers to contact. This caused confusion among the citizens about who to contact in order to receive the help they need, leading to both potentially dangerous situations and inequality of care [1]. Besides, fragmented healthcare systems are more likely to allocate the scarce healthcare resources inefficiently and risk to provide a lower quality of care [2]. In combination with the increasing demand for primary, ambulance, and secondary care services caused by population aging and a higher prevalence of long-term conditions, there became a growing need for a more centrally coordinated system that ensures continuity of care [3, 4].

Therefore, like many other countries, major reforms were initiated in 2014 to create a highly integrated EMS system in Copenhagen. The Regional Council initiated the reforms. The Regional Council is the democratically elected assembly which manages healthcare, both hospital and prehospital, in Copenhagen. A central part of this integrated system was the creation of a new centralized access point in the form of a telephone medical helpline to replace the prior OOH services provided by GPs. The new medical helpline (MH-1813) can be dialed with the number 1813 and is 24 h a day open for any medical advice in non-emergency situations. While the MH-1813 is accessible during GP office hours, the helpline is not a replacement for GP telephone consultations during the daytime. The MH-1813 is staffed with nurses and physicians who can schedule appointments at many different healthcare units, including emergency departments, psychiatric care units, and emergency dentists. In addition, they can send out an OOH GP for a home visit. The dispatchers of the MH-1813 can also access the system of the Emergency Medical Dispatch Center (EMDC-112), which enables them to directly dispatch an ambulance in case a call turns out to require urgent medical help.

To facilitate well-informed triage and enhance patient safety, both the EMDC-112, MH-1813, and the ambulance staff have access to the same electronic patient record. The regional structure, where all hospitals and the emergency medical service are operated by the Regional Council, enables the possibility for shared patient information across hospitals and emergency medical services, where all patient information systems have been standardized. Since the pre-hospital patient record is integrated into this system, the receiving hospital staff can prepare themselves before the patient arrives with real-time information sent from the ambulance [4].

The primary aim of the centralization of the system is to offer greater continuity of care, expand access and choice, and relieve the pressure on hospital emergency departments by the gatekeeping role of the GPs and MH-1813 [3, 5]. The MH-1813 has a central role in providing good triage and avoiding long waiting times at emergency departments by offering patients the opportunity to wait at home instead. In this paper, we will describe the reformed system in more detail, illustrated by the actual numbers in terms of the number of ambulance dispatches and costs both before and after the centralization reforms in 2014.

## Methods

This analysis of the organizational changes is of exploratory and quantitative nature. It focuses on patient flows that entered the healthcare system via the centralized access points for emergency care (EMDC-112) and non-emergency care (MH-1813). For this, annual data both before and after the centralization reforms in 2014 is presented. This paper limits itself to the description of the EMS in the Copenhagen region. There are no data included from other parts of Denmark or other parts of the healthcare system such as internal hospital data.

### Setting

The Danish healthcare system is tax-based and centralized on the regional level [6]. The largest of those regions is the Copenhagen region, covering 1.8 million citizens with 6 hospitals and 8 emergency care units [7]. Emergency calls that come in via the EMDC-112 are triaged via the system of the Danish Index for Emergency Care. This decision tool for follow-up action has five levels, ranging from levels A to E. Level A represents potentially life-threatening conditions and level E non-urgent conditions that do not require a vehicle dispatch. In between, level B represents conditions that are urgent but not life-threatening, level C is not urgent but where transportation and observation are necessary, level D is planned patient transport, and level E refers to not urgent and also no action necessary [7, 8]. The MH-1813 uses a different decision tool, because of the difference in the target group. Yet, the software environment of the two dispatch centers are intertwined, which allows the MH-1813 to quickly transfer calls or immediately dispatch an ambulance if they recognize a caller with an emergency situation. Prior to the organizational change, the GP OOH collaborative did not have the option to directly dispatch ambulances but could contact EMS and request an ambulance [8]. There have been no changes to the staffing of ambulances because of the organizational change.

Besides ambulances, also special vehicles can be dispatched such as the helicopter (HEMS), ambulances equipped for neonatal patients (“babylance”), cars with paramedics specialized in patients with psychological illnesses (“psycholance”), and cars staffed with social workers (“sociolance”) for vulnerable citizens. In case of a (potential) life-threatening condition (dispatch level A and sometimes B), a “mobile critical care unit” (MCCU) car is dispatched in addition to the normal ambulance. This MCCU is staffed by a prehospital physician and a paramedic to support the ambulance crew.

When a cardiac arrest is suspected and quick resuscitation is desired, another system can be activated where citizens are the first responders. This system of “HeartRunners” is based on a smartphone app technology that alarms laypersons in the neighborhood of the patient and asks them to either bring an AED or go directly to the scene to start the resuscitation [9, 10]. Also, patient transport is integrated into the dispatch system and can be booked via an electronic system or via the de dispatch centers. MH-1813 organizes the planned laying patient transport (level D car), and the EMDC-112 is responsible for the unplanned laying inter-hospital transport (ambulance and MCCU).

To improve efficiency in the hospital emergency departments, patients are separated upon arrival according to their healthcare needs. Patients with an, upon triage, unclear disease (e.g., inexplicable abdominal pain) are brought to the diagnosis section and patients with a clear diagnosis (e.g., suspicion of broken bone) are directly brought to the treatment section. To avoid overcrowding of the emergency department waiting rooms, a system has been installed from where MH-1813 triage operators can schedule time slots at the emergency departments for their callers, in order to allow them to wait at home before the appointment.

### Data collection

Where available, internal aggregate data was retrieved from the EMS Copenhagen for the period between January 1, 2010, and December 31, 2019. The EMS Copenhagen changed dispatch systems in December 2013; hence, activity registration methods might slightly differ between years. Descriptions of services are illustrated with current data and where available in comparison with historical data. Data gaps are caused by different payment systems before and after the reforms of 2014. Our investigation is based on the internal registration of dispatch numbers and expenditure of the EMS Copenhagen. As dispatches classified as level E in the Danish Index for Emergency Care refer to calls to the emergency system that require no action or dispatch, these are left out of the analysis.

Before the establishment of the MH-1813, the OOH services were organized by self-employed GPs. Data about those services were not available for evaluation. Data after the 1st of January 2014 were collected from all contacts to the two EMS telephone lines (EMDC-112 and MH-1813). Both electronic decision tools are integrated into the software system “Logis CAD” [11]. Here, the reason for encounter and triage response is electronically registered as incident data. To take into account the fluctuations in population numbers, information about the number of inhabitants in the Copenhagen region was retrieved from Statistics Denmark [12]. To plot the number of dispatches against the expenditure, the annual Consumer Price Index (CPI) in Denmark was retrieved from Statistics Denmark [13], and the average exchange rate between Danish krone (DKK) and euros (€) for the year 2019 was retrieved from the European Central Bank [14].

### Patient and public involvement

There has been no patient and public involvement in this study.

### Analysis

Descriptive analyses were performed, and absolute numbers and percentages for variables were reported. The annual costs of the EMDC-112 and MH-1813 were corrected for inflation using the CPI. First, the baseline year for the expenditure was set on the year 2010, and the expenditures of the succeeding years were adjusted into the actual value of 2010 to keep the buying power the same. Thereafter, the costs in Danish krone were re-calculated into euros using the average exchange rate between the two currencies in the year 2019. To take fluctuations of the number of inhabitants of the region into account, the number of dispatches per response level, expenditure, and the number of calls are presented per 10,000 inhabitants in the respective years.

## Results

Whereas the number of emergency calls to the EMDC-112 stayed more or less the same, the number of calls to the MH-1813 increased from 937,058 in 2015 to 1,008,401 in 2019 (Table 1). Since the incorporation of the GP OOH service into the MH-1813 (2014), the number of GP visits decreased gradually by 51% over the course of 5 years (*n* = 12,670). Despite the increase in level A dispatches between 2014 and 2015, the number of MCCU dispatches dropped by 12% (*n* = 2513). The HEMS was dispatched between 315 and 384 times a year since it was in full operation.

**Table 1:** Number of calls and dispatches in the years before (2010–2013) and after (2014–2019) the reforms

	2010	2011	2012	2013	2014	2015	2016	2017	2018	2019
Calls										
EMDC-112	n.a.	82,838	119,534	118,750	129,886	132,484	133,772	126,059	131,377	130,212
MH-1813	–	–	–	–	–	882,483	914,217	944,789	979,515	1,008,401
MH-1813 waiting time										
Median (s)	–	–	–	–	–	269	232	173	103	108
90 percentile (s)	–	–	–	–	–	996	1128	843	821	755
Median response time by response level (min)										
A	–	6:29	6:15	6:08	6:29	6:32	6:40	6:28	6:38	6:49
B	–	13:51	13:56	14:19	17:45	19:25	18:42	18:44	20:34	20:49
Dispatches by response level^b^										
A	56,692	52,713	52,421	51,773	66,407	67,158	68,031	74,781	84,875	85,393
B	59,189	71,434	72,960	76,657	96,986	93,929	94,053	98,338	99,497	92,097
C	56,260	42,225	34,437	32,125	27,169	26,438	24,341	22,624	21,377	21,954
D	49,283	54,184	60,569	59,048	58,985	57,222	62,948	65,517	67,962	66,526
Acute inter-hospital transport	8216	8525	10,189	10,859	10,974	10,448	10,220	9819	9793	8968
MCCU	13,103	16,411	19,563	19,047	19,369	16,676	16,579	17,990	19,539	17,048
Dispatched cars by type^c^										
Ambulance (yellow, acute)	n.a.	116,397^a^	179,656	178,087	199,838	196,037	199,972	210,751	220,759	213,287
Ambulance (white, planned)	n.a.	43,745^a^	50,240	49,876	49,749	48,735	49,406	50,510	52,953	52,694
MCCU	n.a.	11,821^a^	19,666	19,033	21,730	19,217	19,499	20,564	22,216	19,872
HEMS	–	–	–	–	79	315	359	323	384	381
Babylance	–	–	–	–	–	–	136	254	215	253
Psycholance	–	–	–	–	664	891	931	902	923	789
Sociolance	–	–	–	–	–	32	1294	1686	1882	1896
OOH GP home visits	–	–	–	–	24,868	20,318	15,117	14,900	14,830	12,198

^a^Data from February 11, 2011

^b^Including intrahospital assignments; ambulance only (MCCU, HEMS, babylance, psycholance, sociolance excluded)

^c^Some cars can be dispatched for multiple response levels

**Fig. 1:**
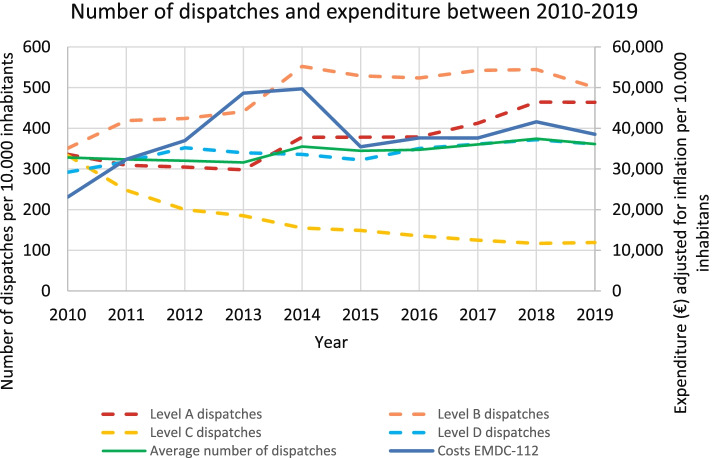
Number of dispatches per category and EMDC-112 expenditure adjusted for inflation between 2014 and 2019

When the number of dispatches is plotted against the population growth, the number of level A dispatches increased with 128 dispatches per 10,000 inhabitants (38%), B increased with 149 (30%), C decreased with 214 (− 64%), and D increased with 69 (24%) (Fig. 1). The average number of dispatches increased from 328 per 10,000 inhabitants in 2010 to 361 in 2019 (10%). Figure 1 also shows the expenditure (in €) of the EMDC-112 per 10,000 inhabitants over the years, adjusted for inflation over time. Before the reforms, the costs increased from €23,096 per 10,000 inhabitants in 2010 to €49,709 in 2014 (115%). Thereafter, it dropped again in the consequent years until €38,523 in 2019 (− 67%).

**Fig. 2:**
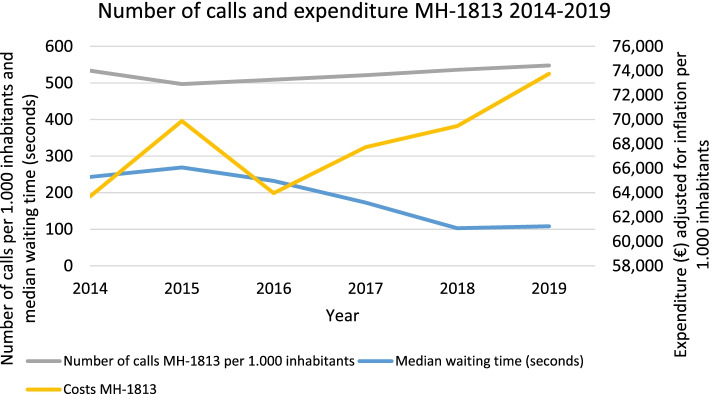
Number of calls, waiting time, and MH-1813 expenditure adjusted for inflation between 2010 and 2019

Figure 2 shows the number of calls to the MH-1813 per 1000 inhabitants and the average waiting time. In the first year of the reform (2014), there were 533 calls per 1000 inhabitants. It dropped to 497 in 2015 (− 7%) and increased thereafter to 548 calls per 1000 inhabitants in 2019. The costs of the MH-1813 per 1000 inhabitants increased from €63,706 in 2014 to €69,882 in 2015 (10%), dropped again in 2016 to €63,965 (− 8%) to where it started and increased again to €73,750 per 1000 inhabitants in 2019 (15%). The median waiting time for callers to the MH-1813 was halved from 243 s in 2014 up to 108 s in 2019.

**Fig. 3:**
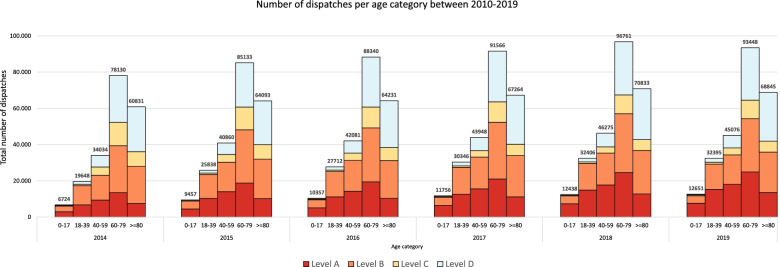
Number of dispatches per age category by urgency level between 2014 and 2019

Figure 3 shows the number of dispatches per age category by urgency level between 2014 and 2019. For every age group, the total number of dispatches increased from 2010 to 2018. Between 2018 and 2019, a drop is seen in the number of dispatches for all adults (≥ 18 years). People aged between 60 and 79 accounted every year for the most dispatches (average: 38%).

## Discussion

In this paper, we described the major reforms in the organization of the regional EMS system around Copenhagen. The system changed from an elaborate incomprehensible system to a patient-centered unified system with clear access points to both emergency and non-emergency medical services. The need for a non-emergency medical helpline in the population was reflected in more than 900,000 calls that were already made in the first year after the launch. Since then, the number of calls increased almost every year, indicating that people are aware of its existence and continue to use it. We believe that the wide variety of dispatch opportunities of MH-1813 allows better coordination of patients and contributes to the continuity of care of patients with healthcare needs both today and in the future. Patient safety in emergency situations is reassured by the synergy between the EMDC-112 and MH-1813, providing immediate help to those who are in need of it.

Over the years, an increase in level A ambulance dispatches was observed, even when adjusted for population growth. This supports the assumption that we can expect more complex healthcare needs in the future as a consequence of the aging of the population and an increased proportion of people dealing with chronic conditions [15]. Level B dispatches increased steadily in the years before the reforms but stabilized after 2014. Level C dispatches dropped gradually from 2010 onwards almost with the same pattern as the increase of level B dispatches. The contribution of every age group to the number of dispatches per urgency category remained stable, which can be expected from a well-functioning triage system. The large drop in EMDC-112 costs was caused by the new tender for operating the ambulance service in the region.

We observed a large fluctuation in costs and waiting times in the first years after the introduction of the MH-1813. It shows that the radical changes in terms of bringing a dispersed non-emergency medical care system together into one central access point required a lot from the organization. The consequent increase in expenditure on the MH-1813 went together with a slight increase in the number of calls and a major reduction in median waiting time. Therefore, we expect that the cost increase was mainly caused by hiring more manpower to deal with more calls and reduce the waiting times simultaneously. Given the large increase in expenditure and reduction in median waiting time, we hypothesize that the marginal costs of reducing the waiting times are high.

This article is limited by the comparability of the data between the years before and after the reforms. Over the years, also the administrative aspects of the system changed along with the system changed, which limits the comparability of the dispatch data. In December 2013, a new computer dispatch system was launched, which could have caused differences in the way the dispatches were counted. Here comes along that the system finds itself in a continuous process of feedback loops and adjustments, which results in gradual changes that happened after the official implementation of the reforms. Another limitation of this paper is that we were not able to evaluate all aspects of the EMS system, such as hospital data on the actual waiting times of the emergency departments and the satisfaction of people who are sent to the emergency department.

The adaptations in the organization of the EMS Copenhagen fit the current trend where many countries reformed their OOH primary care system by piloting, implementing, or improving telephone triage, centralizing the OOH primary care, and changing the organizational model into sustainable high-quality patient-centered care [1, 16, 17]. The goal of such a patient-centered care approach by organizing a central access point to healthcare services is to match the resources and services to the needs of the community and consequently improve the overall public health of the population both somatically and regarding their mental health [18]. Other EMS services have created secondary telephone triage services for low-acuity patients calling 1-1-2 or an equivalent emergency number [19, 20].

Systems similar to the MH-1813 have been piloted or implemented in many other countries. Outcomes of a pilot of the NHS 111 system also showed that the telephone service was also already frequently used in the first year of operation. Also, the observed increase in the numbers of emergency ambulances sent to patients was observed in the NHS 111 system in the first year of operation [17]. The gradual growth of the number of callers was also observed in the NHS 111 system, which strengthens the idea that there is a demand for such a telephone service to access urgent care [21].

The primary role of telephone triage in a system is to ensure that patients are treated at the lowest effective cost level [3]. Studies suggest that telephone advice centers could reduce emergency department activity and GP workload, and the urgency level of the caller is generally estimated correctly [22]. Although there are mixed opinions about using nurses as call-handler [3, 5], studies found no evidence to suggest that physicians are any better or more cost-effective at triage than experienced nurses [23]. As studies show that up to 40% of patients with urgent time-critical conditions (e.g., acute myocardial infarction and stroke) only contact an OOH telephone service, a close collaboration between the EMDC-112 and the OOH telephone service is required [24].

Maintaining such an integrated system will require constant committed executive leadership and a coherent organizational structure, where all participants should share the same values and goals in order to maximize the benefit to the patients [18]. In order to evaluate the performance of those systems, data is required that is not fragmented into distinct silos relating to specific activities or diagnostic groups. Information systems that are capable of reflecting the reality of complex health conditions are fundamental to evaluate the quality and the outcomes of primary care, given the increasing numbers of patients with multiple chronic conditions [3].

## Conclusion

In this paper, we described a newly introduced emergency, non-emergency, and OOH-integrated system in the Copenhagen region. The aim of the new system was to improve the provision of timely and easily accessible care and an efficient distribution of resources. The description was supported with annual dispatch numbers from both before and after the system reforms. Although the transformation process of such an intertwined and complex, we believe that this new system makes clinical sense and is sustainable to also serve its covering population in the future, where complex healthcare demands caused by multi-morbidities are likely to increase.

## Data Availability

Dispatch numbers and expenditures were based on internal aggregate data of the Capital Region of Copenhagen Emergency Medical Services. Data is available upon request at the Copenhagen EMS with approval of the suggested protocol by the executive level. No participants are actively involved in this study.

## Abbreviations

CPI: Consumer Price Index
DKK: Danish krone
EMDC: Emergency Medical Dispatch Center
EMS: Emergency medical services
GP: General practitioner
HEMS: Helicopter emergency medical service
MCCU: Mobile critical care unit
MH: Medical helpline
OOH: Out-of-hours
